# The toxicity of the methylimidazolium ionic liquids, with a focus on M8OI and hepatic effects

**DOI:** 10.1016/j.fct.2019.111069

**Published:** 2020-02

**Authors:** Alistair C. Leitch, Tarek M. Abdelghany, Philip M. Probert, Michael P. Dunn, Stephanie K. Meyer, Jeremy M. Palmer, Martin P. Cooke, Lynsay I. Blake, Katie Morse, Anna K. Rosenmai, Agneta Oskarsson, Lucy Bates, Rodrigo S. Figueiredo, Ibrahim Ibrahim, Colin Wilson, Noha F. Abdelkader, David E. Jones, Peter G. Blain, Matthew C. Wright

**Affiliations:** a Institute of Cellular Medicine, Newcastle University, Newcastle Upon Tyne, NE2 4AA, United Kingdom; b Department of Pharmacology and Toxicology, Faculty of Pharmacy, Cairo University, Kasr El-Aini St., Cairo, 11562, Egypt; c School of Civil Engineering and Geosciences, Drummond Building, Newcastle University, Newcastle Upon Tyne, NE1 7RU, United Kingdom; d Department of Biosciences, Durham University, Durham, DH1 3LE, United Kingdom; e Department of Biomedical Sciences and Veterinary Public Health, Swedish University of Agricultural Sciences, Uppsala, Sweden; f Freeman Hospital, Newcastle Upon Tyne, Tyne and Wear, NE7 7DN, United Kingdom

**Keywords:** PBC, Ionic liquids, Liver, C8[mim], Autoimmunity, Xenobiotics, AhR, aryl hydrocarbon receptor, ALP, alkaline phosphatase, AMA, anti-mitochondrial antibody, AR, androgen receptor, ERα, estrogen receptor α, GGT, γ-glutamyltransferase, M8OI, 1 octyl 3 methylimidazolium, PBC, primary biliary cholangitis, also previously known as primary biliary cirrhosis, PDC-E2, pyruvate dehydrogenase complex E2 component, PPARα, peroxisome proliferator activated receptor alpha

## Abstract

Ionic liquids are a diverse range of charged chemicals with low volatility and often liquids at ambient temperatures. This characteristic has in part lead to them being considered *environmentally-friendly* replacements for existing volatile solvents. However, methylimidazolium ionic liquids are slow to break down in the environment and a recent study at Newcastle detected 1 octyl 3 methylimidazolium (M8OI) – an 8 carbon variant methylimidazolium ionic liquid - in soils in close proximity to a landfill site. The current M8OI toxicity database in cultured mammalian cells, in experimental animal studies and in model indicators of environmental impact are reviewed. Selected analytical data from the Newcastle study suggest the soils in close proximity to the landfill site, an urban soil lacking overt contamination, had variable levels of M8OI. The potential for M8OI - or a structurally related ionic liquid – to trigger primary biliary cholangitis (PBC), an autoimmune liver disease thought to be triggered by an unknown agent(s) in the environment, is reviewed.

## Highlights

• “Ionic liquids” defines a diverse range of chemicals proposed as green chemicals.
• There are limited data in mammalian systems regarding their potential toxicity.
• The ionic liquid M8OI has been found at high levels in the environment.
• M8OI inhibits mitochondrial function and induces mammalian cell apoptosis.
• M8OI is metabolised to a lipoic acid mimetic and may be a hazard trigger for PBC.

## 1 Introduction

Ionic liquids (also frequently referred to as ionic solvents) are salts - normally composed of organic cations and inorganic anions - with a melting temperature below 100 °C and often, they are liquids at ambient temperatures (Wilkes, 2002). Given their relative low volatility, they have been proposed as environmentally-friendly solvents (Wilkes, 2002).

The modern era of ionic liquid development started with the generation of 1-butylpyridinium chloride–aluminium chloride mixtures in the 1970s (Gale et al., 1978; Wilkes, 2002; Welton, 2018). However, they are man-made chemicals that do not exist naturally in biological systems and as a consequence, resist degradation in the environment.

Ionic liquids may currently find use in plant protection products e.g. herbicidal phenoxyacid derivatives. (Pernak et al., 2016). They may also be used in chemical syntheses, biotechnology and chemical engineering - in applications such as cellulose processing, as lubricants, corrosion inhibitors and battery electrolytes, for water purification, chemical separation and extraction and biofuel production (Oskarsson and Wright, 2019).

The ionic liquid 1-octyl-3-methylimidazolium (M8OI, also referred to as C8[mim]) was recently found at high levels in soils in close proximity to a landfill waste site in the North East of England, and to have the potential to trigger an auto-immune liver disease primary biliary cholangitis (PBC) (Probert et al., 2018). M8OI is an 1-alkyl-3-methyl imidazolium ionic liquid, one of the four major chemical classes of ionic liquids that also includes N-alkyl-pyridinium, tetraalkyl-ammonium and tetraalkyl phosphonium liquids (Mallakpour and Dinari, 2012). This article reviews the current state of knowledge regarding the toxic effects of M8OI in mammalian and environmental models and their potential to trigger PBC.

## 2 The toxicology database for M8OI

There are a limited number of studies examining the toxicity of M8OI. Given that toxicity of the cation may be influenced by its anion, the salt used in the studies is identified.

### 2.2 Signalling pathway interactions

A number of bioassays have been performed on M8OI (Cl-salt) and are freely available (https://pubchem.ncbi.nlm.nih.gov/compound/2734223), populated from the Toxicology in the 21st Century (Tox21) program. These data indicate that M8OI interacts with the human sonic hedgehog signaling (Shh); androgen receptor; estrogen receptor alpha; estrogen related receptor; pregnane X receptor and retinoid-related orphan receptor gamma signalling pathways. M8OI is also reported to be a disruptor of mitochondrial membrane potential and to be cytotoxic.

### 2.3 In vitro (mammalian) studies

M8OI (as the Cl^−^ salt) was shown to increase oxidative stress and induce the apoptosis of a human hepatocarcinoma cell line (QGY-7701) by Jing et al. (2013), with an EC_50_ of approximately 360 μM M8IO (as the Br^−^ salt) was also reported to be cytotoxic to the human hepatoma HepG2 cell line but, in agreement with our observations (unpublished), high concentrations - EC_50_ of 439.46 μM after 24 h exposure – were required (Li et al., 2015). An examination of a variety of toxic endpoints suggested that an induction of reactive oxygen species was an early effect of exposure and that the mode of cell death was apoptotic based on induction of caspase activities (Li et al., 2015).

Liu et al. (2016) showed that imidazolium-based ionic liquids (as the Cl^−^ salts) intercalate with DNA isolated from calf thymus, with ionic chain length giving rise to increasing binding strength. Thus, M8OI was shown to be relatively weak at quenching the fluorescence of an ethidium bromide-DNA complex at 666 μM (<10%) whereas a related ionic liquid in which the octyl chain was replaced with a 16 carbon chain at this concentration resulted in >80% fluorescence quenching.

Ma and Li (2018) further investigated the effects of M8OI (as the Br^−^ salt) in HepG2 cells and demonstrated that exposure resulted in changes in heat shock protein 70 and heat shock protein 90 expression and generally inhibited the total anti-oxidative capacity of the cells. There was increased phosphorylation of p53, mitochondrial membrane disruption, cyclooxygenase-2 activation, Bcl-2 family protein modulation, cytochrome *c* and Smac/DIABLO release and inhibition of apoptosis inhibitory protein-2 (c-IAP2) and survivin. The mitochondrial effects and toxicity were partly inhibited by N-acetyl-cysteine.

The liver progenitor B-13 cell line (Probert et al., 2015) was observed to be markedly sensitive to the Cl^−^ salt of M8OI (EC_50_ for MTT reduction ~ 50 μM) and at least 10-fold more sensitive than the hepatocyte-like (B-13/H) cells derived from them (Probert et al., 2018). The earliest effect (within minutes) observed after exposure to M8OI was an inhibition of oxygen consumption in both B-13 and B-13/H cells. This resulted in AMPK phosphorylation and enhanced glucose consumption. Replacing glucose with galactose also sensitized cells to M8OI. Around the time of glucose depletion, the cells underwent apoptosis as evidenced by an induction of caspase 3/7 activity and nucleosomal DNA cleavage.

These data suggest that an interaction of M8OI with mitochondria is likely a key initiating event in the adverse outcome pathway for M8OI toxicity.

### 2.4 Published in vivo (Mammalian) studies

Only one study has been published, to our knowledge, in a mammalian species in vivo. The acute toxic effects of 1-methyl-3-octylimidazolium bromide [M8IO^+^ Br^−^] in mice has been examined. The study was limited to potential adverse effects up to 24 h after an i.p. administration. Ten hours after administration, the authors report histopathological changes in the liver and calculated an LD_50_ of 35.7 mg/kg bw (Yu et al., 2008).

In our hands, the target organ for the toxic effects of M8OI (as the Cl-salt) after i. p. injection was seen to be the kidney based on increases in serum creatinine, urinary protein, urinary kidney injury molecule 1 (Kim1) and histopathological alterations. These effects were clearly evident at doses of 10 mg/kg bw per day (dosed twice over a 24 h period). Effects on the liver at this dose were restricted to depletions of glycogen and portal tract changes in the absence of any overt histopathological changes associated with tissue injury (Leitch et al., manuscript in submission). Given that the proposed mechanism by which M8OI interacts with cells is via an inhibition of oxidative phosphorylation in mitochondria, the kidney may be sensitive to M8OI through its reliance on cellular respiration for reabsorption. Although constituting around 0.5% of body mass, kidneys consume 10% of the oxygen used in cellular respiration (Berg et al., 2002). The kidney may also likely be the primary route for elimination of systemic M8OI and therefore be exposed to high intracellular concentrations of M8OI.

### 2.5 Published studies with model indicators of environmental impact

M8OI [as the Br^−^ salt] has been shown to be acutely toxic to frogs (*R. nigromaculata*) during early embryonic development, with the median lethal concentration values at the early cleavage, early gastrula and neural plate stages of development being 85.1, 43.4, and 42.4 mg/L respectively after 96 h exposure (equivalent to 309, 158 and 154 μM respectively) (Li et al., 2009).

M8OI (as the Cl^−^ salt) reduced MTT reduction activity and replication in *E. coli* (DH5a) suspension cultures that was associated with increased cell membrane permeability, with significant effects seen at the lowest concentration examined of 100 μM (Jing et al., 2014).

M8OI (as the hexafluorophosphate salt) was shown to irreversibly inhibit the germination of wheat seedlings at 4 mg/L (equivalent to 11.8 μM) after 7 days of exposure (Liu et al., 2014). M8OI as either the Cl^−^ or Br^−^ salts were also toxic, the anion reported by the authors to have no impact on toxicity (Liu et al., 2016).

Effects on the growth of green algae (S. obliquus) was examined by Liu et al. (2015) who demonstrated that M8IO and other structurally-related ionic liquids (as the Cl^−^ salts) affected membrane permeability, cell morphology and growth (IC_50_ was determined to be between 0.69 and 0.86 mg/L, equivalent to 3.0–3.7 μM).

Deng et al. (2016) examined the effect of M8OI (as the Br^−^ salt) on the marine diatom (phytoplankton) S. costatum and demonstrated that photosynthesis and cell growth were inhibited, the latter with an EC_50_ of 17.9 and 39.9 mg/L (equivalent to 65 and 145 μM) after 48 and 96 h exposure respectively.

Zhang et al. (2016) indicated that M8OI (as the Br^−^ salt) was genotoxic to planarians (flatworms, using D. japonica) through use of a randomly amplified polymorphic DNA assay. Using this assay, the authors report evidence of a dose-responsive genotoxicity (4, 7 and 9 changes detected) after 1 day exposure at concentrations of 74, 147 and 220 mg/L respectively (equivalent to 270, 530 and 800 μM).

Nan et al. (2016) exposed fish (*P. dabryanus*) to M8OI (as the Cl^−^ salt). After 48 h there was 20% mortality when fish were exposed to 126 mg/L and 100% mortality when exposed to 360 mg/L (equivalent to 550 μM and 1.6 mM respectively). The authors reported that the liver was a target organ with evidence for oxidative stress DNA damage and apoptosis presented.

Three types of M8OI (either as the Cl^−^, Br^−^ or tetrafluoroborate) were examined for their effects on wheat seedlings (up to 800 mg/kg in brown soil) over a 13 day period (Xu et al., 2018). All three ionic liquids inhibited growth (shoot length, root length, pigment content and proline content) of wheat seedlings which was associated with the generation of reactive oxygen species.

M8OI (as the nitrate) significantly reduced the microbial populations (bacteria, fungi and actinomycetes) and diversity at 10.0 mg/kg in brown soil on days 10, 20, 30, and 40 (Zhang et al., 2018).

Silver carp (*Hypophthalmichthys molitrix*) were exposed for 60 days to M8OI (as the Br^−^) at concentrations of 1.09 or 4.38 mg/L (Ma et al., 2018). According to the authors, an elevation in a variety of serum markers and biochemical assays indicated fish organ damage and alterations in brain, gill, intestine, kidney, liver and muscle enzymes suggestive of oxidative stress. Some changes in xenobiotic metabolising enzymes activities (erythromycin-N-demethylase and glutathione S-transferases); increases in inflammatory markers (iNOS, IL-1β, TNF-α and NF-κB), altered levels of TGF-β and apoptosis markers were reported in liver. An additional report focussed on effects in the spleen (Ma et al., 2019).

The effect of M8OI (as either the nitrate or Cl^−^) on marine diatom Phaeodactylum tricornutum growth was examined and was shown to be inhibitory, with 96-h EC_50_ values of 24.0 and 33.6 mg/L respectively (Chen et al., 2019). Photosynthesis was considered by the authors to have been inhibited based on significant decreases of chlorophyll *a* and damage to PSII reaction centres. Content of soluble protein superoxide dismutase and catalase initially increased significantly, likely as an adaptive response prior to decreases in the cultures with higher concentrations of ILs (≥20 mg/L).

The current database on the toxic effects of M8OI in mammalian systems is therefore extremely limited. There is currently only a single limited report published on the effects of acute M8OI exposure in mice. In contrast, the number of studies on the effects of M8OI in model indicators of environmental impact are more extensive. These data overall suggest that if levels accumulate above a variable threshold, adverse effects may be seen in the environment, experimental animals and man.

## 3 Exposure to M8OI

Although M8OI and structurally-related methylimidazolium ionic liquids have toxic effects in cells in vitro, in mice and in a variety of model indicators of environmental impact, there is currently no data to determine whether there is significant contamination of the environment and/or exposure in man to these chemicals.

Interrogation of the UK National Poisons Information Service's (http://www.npis.org/toxbase.html) TOXBASE, which provides information on pharmaceuticals, chemicals (agricultural, household and industrial), plants and animals, suggests that M8OI is not a listed ingredient (Prof Allister Vale, National Poisons Information Service UK, personal communication). M8OI is also not listed as an ingredient in any pesticide in the UK according to the UK Health and Safety Executive COPR Database (https://webcommunities.hse.gov.uk/connect.ti/pesticides/); in any cosmetic in the EU (https://ec.europa.eu/growth/sectors/cosmetics/cosing_en) or in any household product in US (https://hpd.nlm.nih.gov/cgi-bin/household/list?tbl=TblChemicals&alpha=A). However, over 60 methylimidazolium ionic liquids have been registered with the European Chemicals Agency (ECHA), several with toxicity dossiers, suggesting they are being used in the EU such that they do not require to be listed as an ingredient in any product.

### 3.1 ECHA database

The ECHA implements the EU's chemicals legislation and is an extensive source of information on the chemicals manufactured and imported in Europe. In the EU, there is an obligation under the Classification, Labelling and Packaging (CLP) Regulation ((EC) No 1272/2008) that requires manufacturers and importers to submit classification and labelling information for the substances they are placing on the market. This information is publicly available within the C&L Inventory held by ECHA (https://echa.europa.eu/regulations/clp/cl-inventory). Examination of this database indicates that there are currently 5 different M8OI salts pre-registered with ECHA, but since no data on their toxicity have been submitted, their production level by any single producer should be less than 100 tonnes/annum (Dr D Bell, ECHA - personal communication). Interrogating the database for structurally-related methylimidazolium ionic liquids currently shows that 31 ethyl, 22 butyl, 4 hexyl and 2 decyl substituted 3-methylimidazolium ionic salts – in addition to the 5 octyl/M8OI liquids – are listed (see Table 1). Table 1 indicates that a number of shorter chain variants are registered which suggests that these variants are being used industrially. However, in several cases, their production levels are confidential so that there remains uncertainty as to whether there is significant hazard from these chemicals at the current time.

**Table 1:** 1-alkyl-3-methylimidazolium (2C–10C alkyl) ionic liquids registered with ECHA.

#	Chemical	CAS #	EC list #	CLP		Production Levels
				Notified classification and labelling according to CLP criteria - Human Health hazards^a^		
2C (Ethyl)						
1	1-Ethyl-3-methylimidazolium iodide	35935-34-3	609-195-2	Data lacking for all toxicity studies		
2	1-Ethyl-3-methylimidazolium methyl carbonate	251102-25-7	607-553-2	Data lacking for all toxicity studies		
3	1-Ethyl-3-methylimidazolium ethyl Sulfate	342573-75-5	608-962-9	Data lacking for all toxicity studies		
4	1-Ethyl-3-methylimidazolium bis(trifluoromethanesulfonyl)imide	174899-82-2	700-235-5	Acutely toxic orally and dermally but no data available		
5	1-Ethyl-3-methylimidazolium nitrate	143314-14-1	626-456-6	Data lacking for all toxicity studies		
6	1-Ethyl-3-methylimidazolium dibutyl phosphate	869858-84-4	628-587-4	Data lacking for all toxicity studies		
7	1-Ethyl-3-methylimidazolium thiocyanate	331717-63-6	628-857-1	Acutely toxic orally, dermally and by inhalation but no data available		
8	1-Ethyl-3-methylimidazolium L-(+)-lactate	878132-19-5	629-129-6	Data lacking for all toxicity studies		
9	1-Ethyl-3-methylimidazolium methyl sulfate	516474-01-4	629-280-8	Data lacking for all toxicity studies		
10	1-Ethyl-3-methylimidazolium tetrachloroaluminate	80432-05-9	629-638-3	Data lacking for all toxicity studies		
11	1-Ethyl-3-methylimidazolium 1,1,2,2-tetrafluoroethanesulfonate	880084-63-9	663-145-4	Data lacking for all toxicity studies		
12	1-Ethyl-3-Methylimidazolium hydrogen carbonate	947601-94-7	666-647-1	Data lacking for all toxicity studies		
13	1-Ethyl-3-methylimidazolium hexafluorophosphate [for Molten Salt]	155371-19-0	671-175-4	Data lacking for all toxicity studies		
14	1-Ethyl-3-methylimidazolium hydrogen sulfate	412009-61-1	679-296-4	Data lacking or inconclusive		
15	1-Ethyl-3-methylimidazolium p-toluenesulfonate	328090-25-1	680-026-2	Specific target organ – lungs, but no data available		
16	1-Ethyl-3-methylimidazolium 2-(2-methoxyethoxy)ethyl sulfate	790663-77-3	680-032-5	Data lacking or inconclusive		
17	1-Ethyl-3-methylimidazolium tetrachloroferrate	850331-04-3	680-125-0	Data lacking or inconclusive		
18	1-Ethyl-3-methylimidazolium trifluoro(trifluoromethyl)borate	681856-28-0	680-165-9	Data lacking or inconclusive		
19	1-Ethyl-3-methylimidazolium aminoacetate	766537-74-0	684-727-4	Acutely toxic orally but no data available		
20	1-Ethyl-3-methylimidazolium (S)-2-aminopropionate	766537-81-9	684-730-0	Acutely toxic orally but no data available		
21	1-Ethyl-3-methylimidazolium diethyl phosphate	848641-69-0	684-879-1	Acutely toxic orally but no data available		
22	1-Ethyl-3-methylimidazolium dimethylphosphate	945611-27-8	689-585-7	Acutely toxic orally but no data available		
23	1-Ethyl-3-methylimidazolium benzoate	150999-33-0	695-723-7	Registration dossier available https://echa.europa.eu/registration-dossier/-/registered-dossier/23760		0–10 tonnes per annum [1]
24	1-Ethyl-3-methylimidazolium chloride/aluminiumchloride (1:1.5)		939-824-9	Data lacking for all toxicity studies		
25	1-Ethyl-3-methylimidazolium methylcarbonate		939-827-5	Acutely toxic orally, dermally and by inhalation but no data available Specific target organ – CNS, but no data available		
26	1-ethyl-3-methylimidazolium bis(fluorosulfonyl)imide	235789-75-0	825-567-9	Specific target organ – Lung, but no data available		
27	3-ethyl-1-methyl-1H-Imidazolium chloride (1:1)	65039-09-0	613-739-4	Acutely toxic orally. Data lacking or conclusive but not sufficient for classification Registration dossier available https://echa.europa.eu/registration-dossier/-/registered-dossier/16488		Tonnage Data Confidential [2]
28	3-ethyl-1-methyl-1H-Imidazolium salt with N-cyanocyanamide (1:1)	370865-89-7	609-330-5	Data lacking or conclusive but not sufficient for classification Registration dossier available https://echa.europa.eu/registration-dossier/-/registered-dossier/21257		10–100 tonnes per annum [3]
29	3-Ethyl-1-methyl-1H-imidazolium 1,1,1-trifluoromethanesulfonate (1:1)	145022-44-2	680-002-1	Data lacking or inconclusive Registration document available https://echa.europa.eu/registration-dossier/-/registered-dossier/12728		0–10 tonnes per annum [4]
30	1-ethyl-3-methyl-1H-imidazol-3-ium-acetate	143314-17-4	604-344-8	Data lacking or inconclusive. Urgent need for toxicological assessment before it can be used in numerous technologies (Ostadjoo et al., 2018). Registration document available https://echa.europa.eu/registration-dossier/-/registered-dossier/25928/7/3/2		0–10 tonnes per annum https://proionic.com/news/reach-registration-EMIM-OAc.php
31	1-ethyl-3-methyl-1H-imidazol-3-ium tetrafluoroborate	143314-16-3	671-177-5	Data lacing or conclusive but not sufficient for classification. Registration document available https://echa.europa.eu/registration-dossier/-/registered-dossier/6591		10–100 tonnes per annum
4C (Butyl)						
1	1-Butyl-3-methylimidazolium methyl carbonate solution	916850-37-8	618-781-7		Acutely toxic orally, dermally and by inhalation. Data often lacking or conclusive but not sufficient for classification. Toxic to all organs.	
2	1-Butyl-3-methylimidazolium bis(trifluoromethanesulfonyl)imide	174899-83-3	605-742-4		Acutely toxic orally and dermally. Data lacking or incoclusive. Toxic to all organs. Toxic to CNS.	
3	1-Butyl-3-methylimidazolium bromide	85100-77-2	617-674-2		Data lacking or inconclusive.	
4	1-Butyl-3-methylimidazolium methyl sulfate	401788-98-5	609-790-7		Data lacking for all toxicity studies.	
5	1-Butyl-3-methylimidazolium iodide	65039-05-6	619-502-1		Data lacking or inconclusive.	
6	1-Butyl-3-methylimidazolium dibutyl phosphate	663199-28-8	628-122-5		Specific target organ toxicity – not identified, no data available.	
7	1-Butyl-3-methylimidazolium dicyanamide	448245-52-1	628-456-1		Acutely toxic orally. Specific target organ toxicity – not identified, no data available.	
8	1-Butyl-3-methylimidazolium trifluoroacetate	174899-94-6	628-479-7		Specific target organ toxicity – not identified, no data available.	
9	1-Butyl-3-methylimidazolium nitrate	179075-88-8	629-008-8		Specific target organ toxicity – not identified, no data available.	
10	1-Butyl-3-methylimidazolium hydrogen carbonate	366491-15-8	629-216-9		Specific target organ toxicity – not identified, no data available.	
11	1-Butyl-3-methylimidazolium tetrafluoroborate	174501-65-6	638-831-1		Acutely toxic orally. Specific target organ toxicity – lungs, no data available.	
12	1-Butyl-3-methylimidazolium trifluoromethanesulfonate	174899-66-2	678-094-3		Acutely toxic orally. Specific target organ toxicity –lungs, no data available. Data lacking or conclusive but not sufficient for classification.	
13	1-butyl-3-methylimidazolium tetrachloroferrate	359845-21-9	678-175-3		Data lacking or inconclusive.	
14	1-Butyl-3-methylimidazolium methanesulfonate	342789-81-5	679-383-7		Acutely toxic orally.	
15	1-Butyl-3-methylimidazolium trifluoro(trifluoromethyl)borate	741677-68-9	679-580-8		Data lacking or inconclusive.	
16	1-Butyl-3-methylimidazolium thiocyanate	344790-87-0	682-764-0		Acutely toxic orally, dermally and by inhalation but no data available. Specific target organ toxicity – not identified, no data available.	
17	1-Butyl-3-methylimidazolium hydrogen sulfate	262297-13-2	684-205-6		Data lacking for all toxicity studies.	
18	1-Butyl-3-methylimidazolium tetrachloroaluminate	80432-09-3	684-907-2		Data lacking for all toxicity studies.	
19	1-Butyl-3-methylimidazolium tribromide	820965-08-0	691-470-1		Data lacking or inclonclusive.	
20	1-Butyl-3-methylimidazolium chloride/aluminiumchloride (1:1.5)		939-823-3		Data lacking for all toxicity studies.	
21	1-Butyl-3-methylimidazolium methylcarbonate		939-828-0		Acutely toxic orally, dermally and by inhalation but no data available. Specific target organ toxicity – CNS, no data available.	
22	3-butyl-1-methyl-1H-imidazol-3-ium chloride	79917-90-1	460-120-8		Registration dossier available https://echa.europa.eu/registration-dossier/-/registered-dossier/12929 This ionic liquid has also been reviewed by the NTP (2004).	Tonnage Data Confidential [5].
6C (Hexyl)						
1	1-Hexyl-3-methylimidazolium trifluoromethanesulfonate	460345-16-8	628-457-7		Specific target organ toxicity – not identified, no data available	
2	1-Hexyl-3-methylimidazolium hexafluorophosphate	304680-35-1	629-544-2		Specific target organ toxicity – not identified, no data available	
3	1-Hexyl-3-methylimidazolium tetrafluoroborate	244193-50-8	680-100-4		Acutely toxic orally but no data available, data lacking or inconclusive	
4	1-Hexyl-3-methylimidazolium chloride	171058-17-6	690-853-0		Data lacking toxicity studies	
8C (Octyl) i.e. M8OI						
1	1-Methyl-3-n-octylimidazolium hexafluorophosphate	304680-36-2	627-887-2		Specific target organ toxicity – not identified, no data available	
2	1-Methyl-3-n-octylimidazolium trifluoromethanesulfonate	403842-84-2	627-928-4		Specific target organ toxicity – not identified, no data available	
3	1-Methyl-3-n-octylimidazolium chloride	64697-40-1	629-637-8		Specific target organ toxicity – not identified, no data available	
4	1-Methyl-3-n-octylimidazolium bromide	61545-99-1	677-551-4		Data lacking or conclusive but not sufficient for classification Specific target organ – Lung, but no data available	
5	1-Methyl-3-n-octylimidazolium tetrafluoroborate	244193-52-0	801-285-1		Data lacking for all toxicity studies	
10C (Decyl)						
1	1-Decyl-3-methylimidazolium tetrafluoroborate	244193-56-4	627-902-2		Specific target organ toxicity – not identified, no data available	
2	1-Decyl-3-methylimidazolium chloride	171058-18-7	629-293-9		Specific target organ toxicity – not identified, no data available	

a Skin Corrosion/Irritation (H315) and Serious Eye Damage/Eye Irritation (H318) are common hazard warnings for these ionic liquids though no data are included (and therefore not taken into consideration). These hazards are likely flagged from read-across considerations. Therefore this category refers to Acute Toxicity – Oral; Acute Toxicity – Dermal; Acute Toxicity – Inhalation; Respiratory Sensitisation; Skin Sensitisation; Aspiration Hazard; Germ Cell Mutagenicity; Germ Cell Mutagenicity; Carcinogenicity; Reproductive Toxicity; Effects on or via Lactation; Specific target organ toxicity; Affected Organs and Specific target organ toxicity – Repeated. [1] This substance is used in the following products: adhesives and sealants, coating products and inks and toners. This substance is used in the following activities or processes at workplace: transfer of chemicals, closed processes with no likelihood of exposure, closed, continuous processes with occasional controlled exposure, closed batch processing in synthesis or formulation, batch processing in synthesis or formulation with opportunity for exposure, mixing in open batch processes, transfer of substance into small containers, roller or brushing applications, non-industrial spraying and treatment of articles by dipping and pouring. Other release to the environment of this substance is likely to occur from: indoor use and outdoor use resulting in inclusion into or onto a materials (e.g. binding agent in paints and coatings or adhesives). [2] This substance is used in the following activities or processes at workplace: transfer of chemicals, closed processes with no likelihood of exposure, closed, continuous processes with occasional controlled exposure, closed batch processing in synthesis or formulation and laboratory work. Release to the environment of this substance can occur from industrial use: for thermoplastic manufacture. [3] This substance is used in the following activities or processes at workplace: closed processes with no likelihood of exposure, closed, continuous processes with occasional controlled exposure, closed batch processing in synthesis or formulation, batch processing in synthesis or formulation with opportunity for exposure and mixing in open batch processes. Release to the environment of this substance can occur from industrial use: formulation of mixtures and formulation in materials. Release to the environment of this substance can occur from industrial use: in processing aids at industrial sites. [4] No information available on it use. [5] No information available on its use.

### 3.2 Discovery of M8OI in the environment

**Fig. 1:**
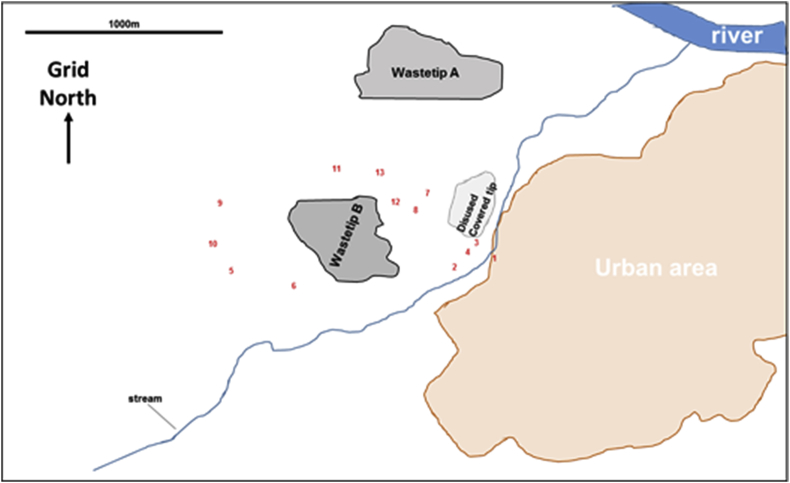
**Approximate location of sampling sites around the landfill waste site used in**Probert et al (2018). Prevailing wind is westerly/south westerly.

The Newcastle study was primarily set up to determine proof of concept whether extracts from soil samples may be screened for the presence of xenobiotics that interact with receptors known to respond to xenobiotics or are toxic to cells. To maximise the potential value of the pilot study, samples were taken from an area in the north east of England that was of potential interest with regard to PBC incidence. The area contained a landfill site and thirteen sites (from allotments, footpaths and the roadside verges) surrounding the peri-urban landfill site were sampled (see Fig. 1).

In accordance with Article 6 of EU directive 199/31/EC on the landfill of waste, the site was (and remains) designated as a non-hazardous waste site. As a non-hazardous waste site, it should only be used for municipal waste, non-hazardous waste of any other origin which meets the criteria set out on Annex II of the directive and stabilized, non-reactive hazardous waste. The landfill site was originally an abandoned stone quarry that operated from the 1930s to the 1960s. It has been used as a landfill site since the late 1990s. The landfill sampling locations were also adjacent to a disused colliery and associated light railway (that operated between 1890 and 1960), remediated in 1990 and converted into public open space. Other land uses in the area have been semi-rural and residential throughout the history of the site. Three separate control sites were also sampled for comparison (from the University farm in rural Northumberland with a controlled fertiliser regime for the last 130 years and from two gardens from separate urban areas in the region). Thus, all sample sites (near landfill and control) were from a region that has experienced a long period of urban growth and industrialisation with particular emphasis on heavy industries such as steel, ship building and coal mining. These industries have dwindled in the last 30 years and the area around the landfill is currently classified as residential and green space (Generalised Land Use Database – Office of National Statistics, 2005). However, the region has been left with a legacy of industrial contamination associated with these industries, specifically toxic elements and polyaromatic hydrocarbons (PAHs). The north east of England is not unusual in having this, and most of the urban areas of the UK share this legacy (Johnson et al., 2012).

Chemicals present in soil samples were extracted into aqueous, ethanol and chloroform liquids (for full methodological details, see Probert et al., 2018). Mammalian cells in culture were initially exposed to these extracts (up to 1% v/v; chloroform extracts were dried and re-suspended in an equivalent volume of DMSO) to screen for toxic effects (e.g. primarily using the MTT screening assay) over several days. There was no apparent microbial contamination of the treated mammalian cell cultures leading to toxic effects (e.g. associated with microbial growth) over the timescale employed, despite the origin of the extracts (Probert et al., 2018). Cells were then transfected with promoter-reporter gene constructs (up to 0.1% v/v, below levels that caused toxicity; chloroform extracts were dried and re-suspended in an equivalent volume of DMSO) as a screen for the potential presence of chemicals activating the aryl hydrocarbon receptor (AhR), pregnane X receptor (PXR), peroxisome proliferator activated receptor alpha (PPARα), estrogen receptor (ER) and androgen receptor (AR). This approach was designed to screen for chemicals capable of activating or inhibiting the receptors. In addition, established assays for environmental contaminants were performed. Elements (As, B, Ba, Cd, Co, Cr, Cu, Pb, Mn, Hg, Mo, Ni, Se and Zn) were determined in soil directly via *aqua regia* digestion by inductively coupled plasma optical emission spectrometry (ICP-OES). The chloroform extracts were analysed for PAHs by GC-MS analysis. Pesticide analysis was determined by UPLC® coupled with Xevo TQ-S (Waters). For full methodological details, see Probert et al. (2018).

**Fig. 2:**
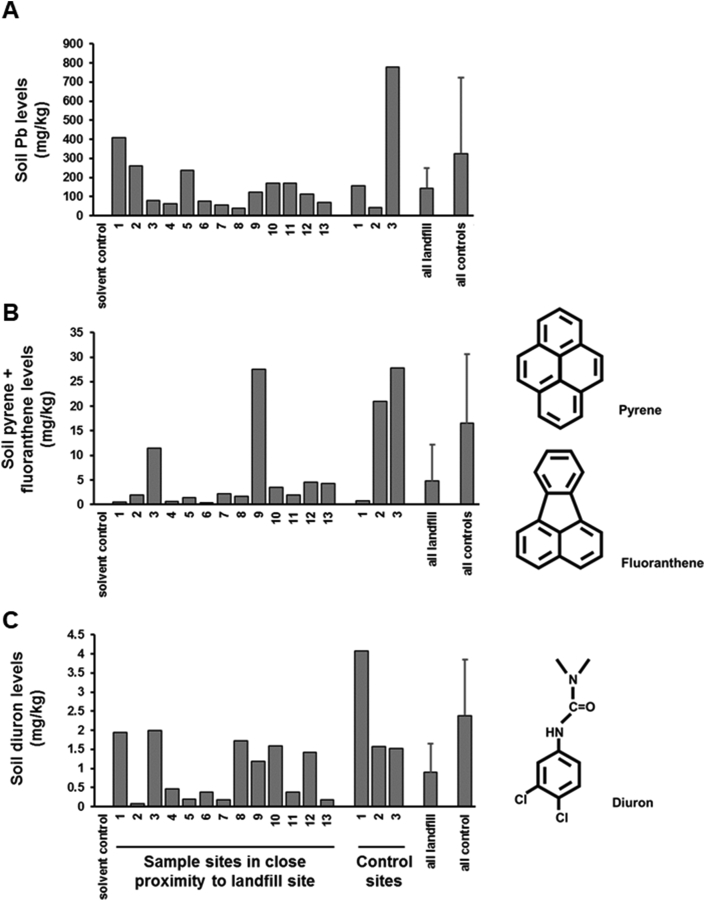
**Concentrations of one toxic element (lead), two PAHs (pyrene + fluoanthene) and one pesticide (diuron) around a landfill waste site and control sites.** Soil samples were processed as outlined (Probert et al., 2018). **A**, Lead (Pb) levels were determined directly *via aqua regia* digestion by ICP-OES; **B**, pyrene and fluoranthene levels in chloroform extracts by GC-MS analysis and **C**, Diuron levels by UPLC coupled with Xevo TQ-S as described (Probert et al., 2018). Sample sites correspond to those schematically described in Fig. 2. All landfill and all control refer to the mean and SD of all sample sites in close proximity to the landfill site and control sites respectively.

The levels of 14 toxic heavy metals (e.g. lead, see Fig. 2a) were determined (Probert et al., 2018). As can be seen for lead levels alone, whilst not significantly different, mean levels were higher for control sample sites than those for sites in close proximity to the landfill site, due to one control site with a very high lead concentration. Considering all 15 toxic elements combined, control sample site mean total levels were 2.0±1.70 g toxic elements/kg soil compared to 2.3±3.47 g toxic elements/kg soil for sample sites in close proximity to the landfill site. Two of the sample sites and two of the control sites had elevated of PAHs. Ten individual categories of PAHs (such as pyrene and fluoranthene, see Fig. 2b) were examined and mean total PAHs were higher in control soils (65 ± 54 mg/kg soil) compared to landfill soils (13 ± 19 mg/kg soil). The concentrations of 24 different pesticides was also examined. Only 2 pesticides (e.g. Diuron, see Fig. 2c and Omethoate) were detected at levels above 1 mg/kg soil. The combined mean pesticide levels were higher in control soils (total of 3.9±1.81 mg/kg soil) compared to soils in close proximity to the landfill site (total of 2.0±1.69 mg/kg soil). Overall, in both control sites and sites in close proximity to the landfill site, contamination with toxic elements, PAHs and pesticides were within normal ranges of an urban soil lacking overt contamination.

All soil sample extracts assessed for activation of selected xenobiotic receptors were initially screened and used at non-toxic levels to reduce the potential for toxicity masking xenobiotic activity on the receptors. Cell viability was primarily assessed using MTT reduction (in conjunction with morphological examination and limited trypan blue dye exclusion tests) since it is amenable to high throughput screening, allowing also for multiple replicates and timepoints. MTT is a substrate for cellular NAD(P)H-dependent oxidoreductase enzymes and its reduction provides an estimate of cellular NAD(P)H generation over a defined incubation period (Berridge and Tan, 1993). MTT reduction assays are widely used as a proxy for cell viability. In all but two extracts (aqueous extracts from sampling sites 1 and 2 in close proximity to the land fill site), there was little evidence for marked reductions in MTT activity. In any case, a control construct (not containing the xenobiotic receptor enhancer sequences) was transfected with the reporter gene construct at a fixed ratio to control for any non-specific (e.g. toxic) effects of extracts on cells. Therefore, reporter gene data were likely solely reflective of receptor activation.

Activation of receptors known to be contacted or activated by xenobiotics (AhR, PXR, PPARα, ER, AR) were used to determine the presence of chemicals activating or inhibiting these receptors. In addition, induction of the nuclear factor erythroid 2-related factor 2 (Nrf2) was examined, as an indicator of oxidative stress. For all these bioassays, except Nrf2, there were chemicals with activity present in both control sites and sites in close proximity to the landfill sites (see Table 2).

**Table 2:** Analytical data and toxicity-related endpoint data from the soil samples studied as described by Probert et al. (2018).

	Control sample sites	Sample sites in close proximity to a landfill site
Analytical		
Pb	326 ± 396 mg/kg soil	144 ± 105
All screened heavy metals	2080 ± 3436 mg/kg soil	1830 ± 1626
All screened PAHs	65 ± 54 mg/kg soil	13 ± 9.0
All screened pesticides	3.9±1.81 mg/kg soil	2.0 ± 1.69
Cell-based toxicity-related endpoint		
Human AhR activation	yes	yes
Human PXR activation	yes	yes
Human PPARα activation	yes	yes
Human ERα activation	no	yes
Human AR activation	low	low
Rat liver progenitor cell toxicity	no	yes - selected

**Fig. 3:**
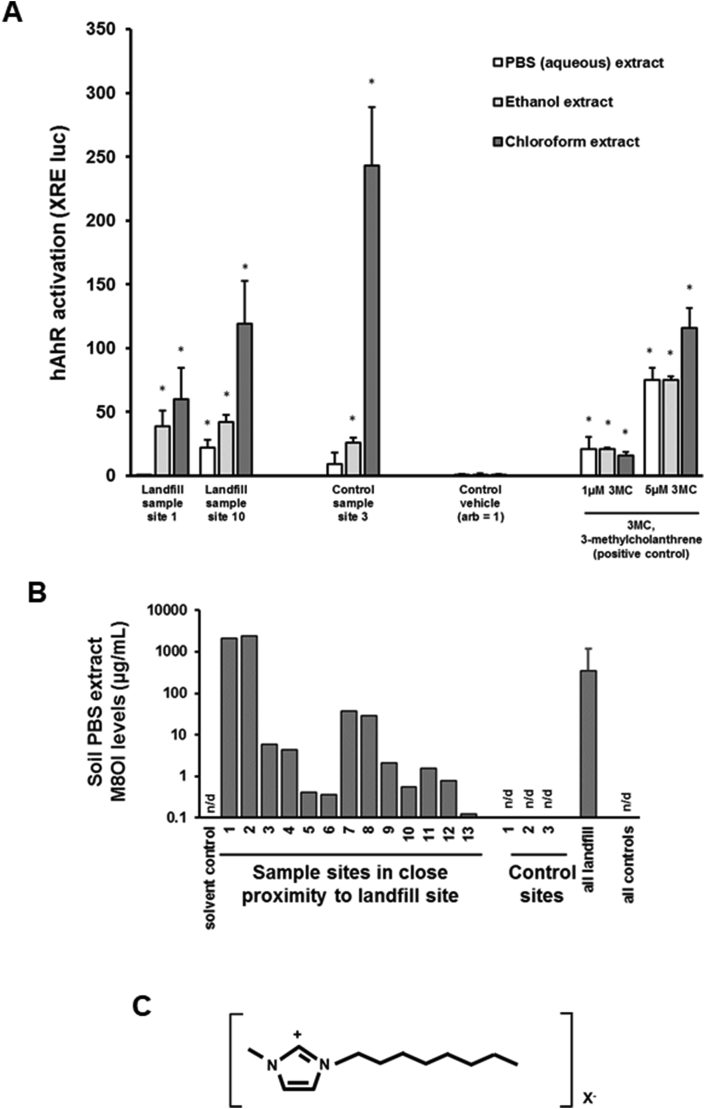
**Effects of extraction liquid on AhR activating activity and concentrations of M8OI from soils**. **A**, human AhR activation (XRE-luc reporter gene) activities in HepG2 cells treated with 0.1% (v/v) of the indicated landfill or control site soil PBS (open bars), ethanol (grey bars) or chloroform extracts (dark grey bars), as outlined in Probert et al. (2018). Data are expressed as fold control vehicles (set arbitrarily to 1) and are the mean and SD of 3 separate determinations. 3 methylcholanthrene is a transcriptional activator of the human AhR (Harvey et al., 2000). **B**, Soil PBS extract M8OI levels, determined and quantified using non targeted data independent LC-HR-MS/MS as outlined in Probert et al. (2018) using authentic pure M8OI as standard. Landfill sample sites 1 and 2 had the highest concentrations of M8OI in the PBS extract (~13 mM), which suggests soil concentrations of M8OI in the region of at least 0.3 ppm. **C**, chemical structure of the M8OI cation.

The AhR is typically activated by polyaromatic hydrocarbons which are generally hydrophobic in nature. It could be seen from any one particular soil sampling site that the activation of the human AhR was highest in chloroform extracts, followed by ethanol and then aqueous extracts (Fig. 3a). In contrast, activation of the nuclear factor-like 2 (Nrf2-Keap1) regulatory pathway was not observed (unpublished data). A number of other reporter gene-based systems were also examined, including the metallothionein response (Probert et al., 2018) and the endoplasmic reticulum stress response (unpublished). Overall, based on receptor-reporter gene expression screening, the only difference between control soils and soils in close proximity to the landfill site was the presence in several landfill sampling sites of human ERα-activating chemicals (Meyer et al., 2017a, Meyer et al., 2017b; Probert et al., 2018).

Based on studies using the murine ER orthologues (cloned for use in another project in the laboratory), it was shown that the murine ERβ variant 2 receptor was “super-activated” by the soil-derived (xeno)estrogens (Meyer et al., 2017a, 2017b), in that activation was not inhibited by the ER antagonist IC 182780 (although endogenous estrogen activation was sensitive to antagonism). M8OI was shown to be capable of this property on the murine receptor however, it did not activate the human ERβ (in HEK293 cells) (Leitch et al., 2018). The implications of ERβ super-activation might have on cholestasis in the mouse and its relevance, if any, in man are currently unknown. However, an agonist for the ERβ has been reported to inhibit liver steatosis in mice fed either a high fat diet or a methionine choline-deficient diet (Ponnusamy et al., 2017).

A number of other hepatic cell lines and primary cell cultures (e.g. human liver cholangiocytes) were also examined given its part-focus on PBC. The B-13 cell line was shown to be particularly-sensitive to chemicals present in the aqueous extract of 2 of the 13 sampling sites in close proximity to a landfill site (an effect absent from all 3 control sampling sites), subsequently shown to contain high levels of (see Fig. 3b), and to be caused by, M8OI (for structure, see Fig. 3c) (Probert et al., 2018). The B-13 cell line is a rodent pancreatohepatobiliary progenitor cell line (it expresses several exocrine pancreatic marker genes in its proliferative B-13 phenotype) that has the unusual property of differentiating into hepatocytes (B-13/H cells) and potentially cholangiocytes (bile duct epithelial cells) in response to a relatively simple hormonal stimulus (Wallace et al., 2010; Wallace et al., 2011, for review of B-13 cells see Probert et al., 2015). It was subsequently shown that M8OI targets the mitochondria in B-13 and human cholangiocytes, resulting in an inhibition of oxidative phosphorylation and loss of ATP levels (Probert et al., 2018). The B-13 cells primarily underwent apoptosis although this ultimately collapses to a necrotic process with time in vitro.

## 4 PBC and M8OI

### 4.1 PBC

**Fig. 4:**
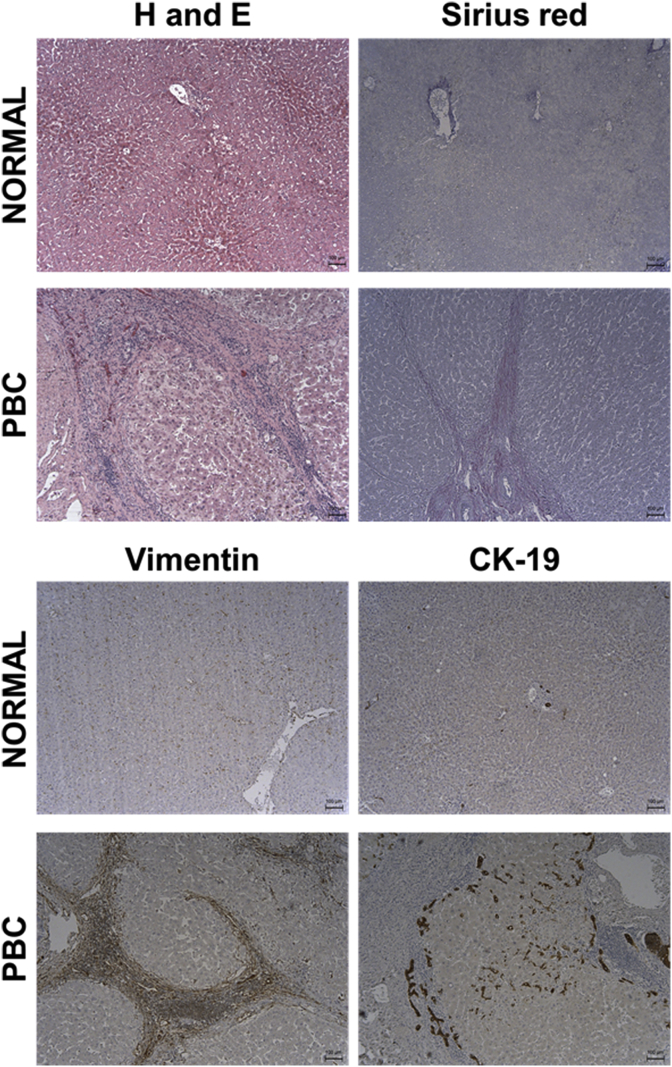
**The pathology of PBC in human liver.** Typical (immuno)histopathology seen in normal human liver and in liver from a patient diagnosed with PBC. Liver tissue was obtained via the Newcastle Biobank (https://www.ncl.ac.uk/biobanks/) with over-arching ethical approval from the Newcastle & North Tyneside 1 Research Ethics Committee. Liver tissue was formalin-fixed, processed and (immuno)stained as indicated essentially as previously outlined (Probert et al., 2014). Sirius red staining identifies collagenous matrix proteins; vimentin identifies fibrogenic fibroblasts typically predominant in a cholestatic liver injury and cytokeratin 19 (CK-19) identifies bile duct epithelial cells (cholangiocytes), for review, see also Wallace et al. (2008).

PBC is a liver disease characterised by an inflammatory and immunoreactive reaction in the portal tract regions of the liver lobule (See Fig. 4). Pathologically, there may be evidence of biliary hyperplasia (suggestive of cholestatic injury) that, presumably with progression, leads to cholangiopathy, loss of intra-hepatic bile ducts and fibrosis (Hirschfield et al., 2018). In the absence of liver pathology being available, unexplained repeated elevations of serum alkaline phosphatase (ALP) and/or γ-glutamyltransferase (GGT) – suggestive of cholestasis - and high titres of anti-mitochondrial antibody (AMA, > 1:40) are considered sufficiently diagnostic for the disease (Hirschfield et al., 2018). PBC occurs more commonly in women than men (approx. 10:1) and most often in post-menopausal women. Although treatments are available to help manage disease progression, there is currently no cure for PBC.

The presence of AMA in >95% of patients has driven the conclusion that PBC is an auto-immune disease, with AMA being immunoreactive to the lipoylated domains of 2 oxo acid dehydrogenase enzymes such as the E2 component of the pyruvate dehydrogenase complex (PDC-E2) (Dyson et al., 2015). AMA-negative PBC is associated with antibodies to other antigens (Hirschfield et al., 2018). Exposure in susceptible individuals to a xenobiotic(s) that mimics lipoic acid and is capable of replacing this factor in lipoylated proteins is one of several theories proposed as a trigger mechanism for PBC. The source of such a xenobiotic(s) could be the environment through pollution (e.g. Superfund chemicals) and/or associated with anthropogenic activities (e.g. release of coal-derived chemicals). These proposals are derived in part from a reported 10-fold increase in PBC incidence when patients obtained drinking water from one particular water reservoir in the Sheffield (UK) region in the 1980s (Triger, 1980); that there is spatial clustering for PBC incidence in the North East of England (which has been intensively mined for coal in the past) (Prince et al., 2001) and that there is an increased prevalence of PBC near Superfund toxic waste sites in the New York City area (Ala et al., 2006).

It should be noted that lipoic acid is an endogenously-synthesised antioxidant (Tibullo et al., 2017) and has been shown to protect cells from the redox active toxins such as arsenic (Samuel et al., 2005) and other heavy metals (Zou et al., 2015; Yang et al., 2017) as well as drugs (Sandhya et al., 1995; Kim et al., 2014; El-Sayed et al., 2017) and chemicals (El-Beshbishy et al., 2014; Aly et al., 2016).

### 4.2 M8OI is metabolised to a lipoic acid-mimicking product by human hepatocytes

As described above, AMA is immunoreactive to the lipoylated domains of 2 oxo acid dehydrogenase enzymes such as the E2 component of the pyruvate dehydrogenase complex (PDC-E2). M8OI bears structural similarity to lipoic acid and the alkyl chain may also be a target for omega hydroxylation (as occurs with fatty acids via cytochrome P450 4As (Misra and Reddy, 2014). Such an hydroxylation on M8OI would be the first step in its conversion to a carboxyl containing metabolite that could render it susceptible to covalent incorporation into 2 oxo acid dehydrogenase enzymes in place of lipoic acid.

**Fig. 5:**
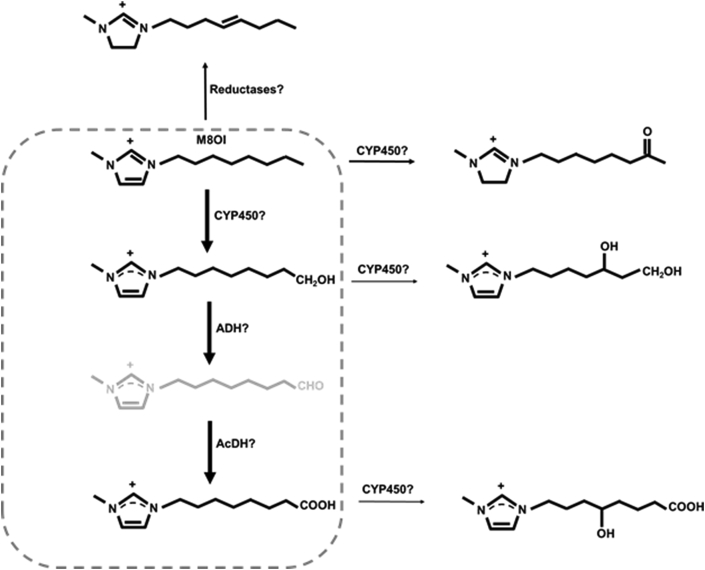
**Proposed metabolism of M8OI by human liver**. Based on data from Leitch et al. (2018). Metabolites within dotted line represent the major human liver metabolites of M8OI.

Incubating human hepatocytes with M8OI resulted in its metabolism to both the omega hydroxylated product 1-(8-hydroxyoctyl)-3-methyl-imidazolium (HO8IM) and further oxidation to the carboxylated product 1-(7-carboxyheptyl)-3-methyl-1H-imidazol-3-ium (COOH7IM) although this was variable, depending on the donor (Probert et al., 2018). Low levels of additional metabolites were also detected that suggest other minor pathways of metabolism may also occur (Fig. 5).

### 4.3 Enzymatic incorporation of COOH7IM into PDC-E2 in place of lipoic acid

**Fig. 6:**
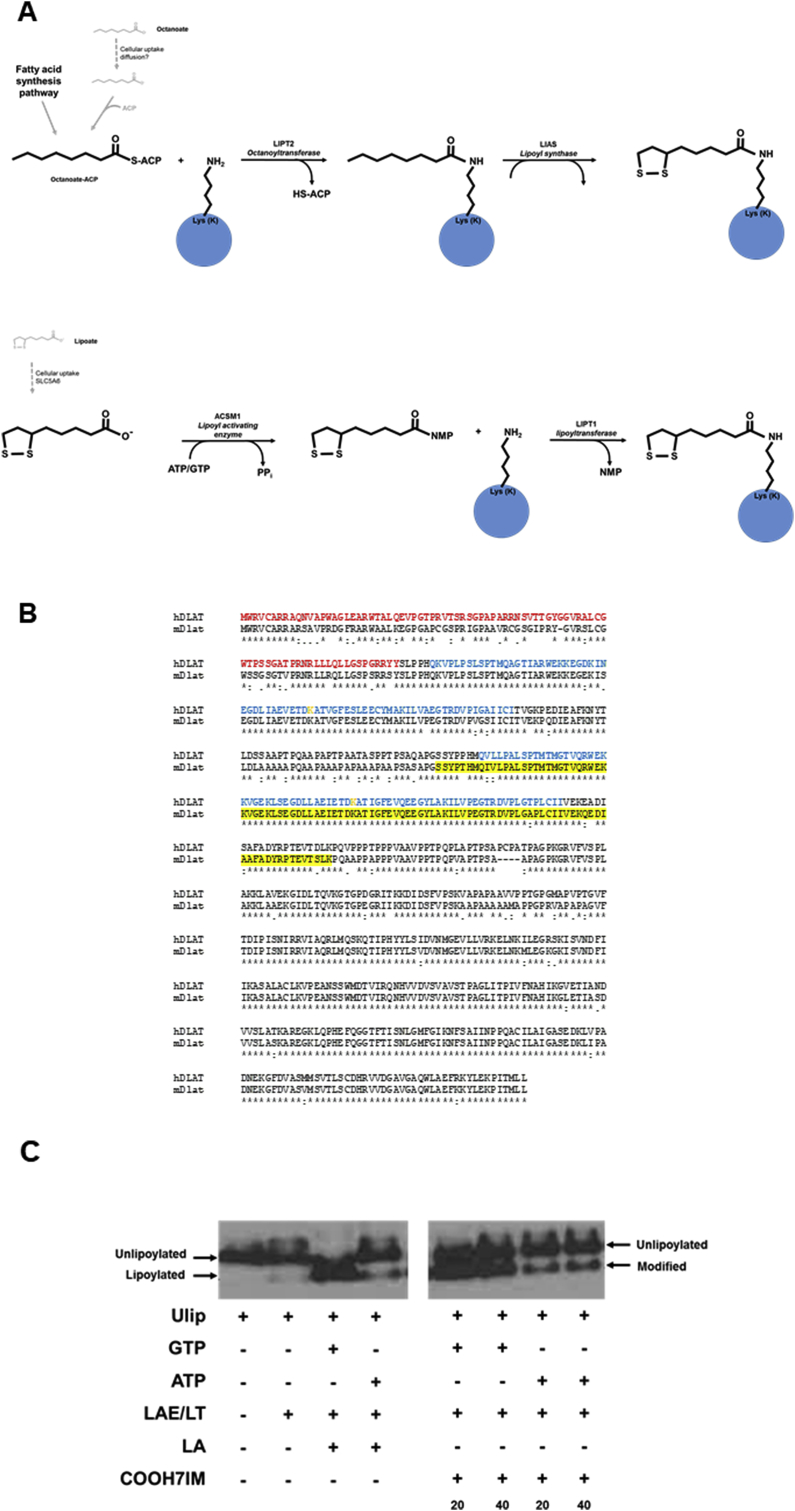
**The conjugation of the E2 sub-unit of PDC. A,** schematic pathway for the lipoylation of proteins in mammalian cells (upper panel) and proposed *xenobiotic scavenging pathway* conjugation of E2 by xenobiotics (lower panel). **B**, alignment of the human PDC-E2 (hDLAT) and murine Pdc-e2 (mDlat) amino acid sequences using CLUSTAL O(1.2.4) multiple sequence alignment. Transit peptide sequence is shown in red; lipoyl domain sequence is given in blue; the lysine (K) residues conjugated with lipoic acid is shown in orange; the yellow highlighted site is the recombinant murine peptide sequence used in the enzymatic lipoylation/xenobiotic conjugation assay, this peptide is flanked at the N-terminus with the sequence ASMTGGQQMGRIRIRAR and at the C terminus with the His tag sequence LEHHHHHH, derived from the expression plasmid used. **C**, Western blot for the detection of unlipoylated, lipoylated or modified Ulip peptide after addition of lipoic acid (LA) or COOH7IM, essentially as outlined in Probert et al. (2018). Ulip, unlipoylated recombinant murine peptide sequence used in the enzymatic lipoylation/xenobiotic conjugation assay; LAE/LT, recombinant bovine lipoate activating enzyme (LAE) and lipoyl-AMP(GMP):N-lysine lipoyl transferase (LT), i.e. bovine Acsm1 and Lipt1.

Lipoic acid is an endogenously produced sulphur-containing molecule required by 2 keto acid dehydrogenase enzymes for their catalytic function (Solmonson and DeBerardinis, 2018). Lipoic acid is covalently conjugated to specific lysine residues, e.g. K132 and K259 in the human E2 component of the pyruvate dehydrogenase complex. In mammals, this occurs indirectly from octanoate via an enzymatic conversion as outlined (Fig. 6a).

As observed in the *E. coli* scavenging pathway, lipoic acid – at least in vitro - may be directly conjugated (in E coli by lipoate ligase LplA) via its activation by the mitochondrial medium-chain acyl-CoA synthetase (Acsm1). However, since exogenous lipoic acid fails to rescue defects in cells derived from lipoylsynthase (LIAS)-deficient patients; Lias-deficiency in mice is embryo lethal and there is no substantial evidence to show that ACSM1 activates lipoic acid in vivo (Solmonson and DeBerardinis, 2018), this “*scavenging pathway*” may only be relevant for xenobiotics.

Using a recombinant peptide sequence consisting of residues 221–314 of the murine E2 component of PDC (Fig. 6b), the enzymatic conjugation of lipoic acid via the *xenobiotic scavenging pathway* in vitro may be followed, through changes in electrophoretic mobility under native conditions using recombinant bovine Acsm1 and Lipt1 enzymes in vitro (Walden et al., 2008). When lipoic acid was replaced by COOH7IM, similar changes in peptide mobility occurred (Probert et al., 2018) indicating COOH7IM conjugation to the peptide and potential conjugation in vivo. Conjugation occurs whether GTP or ATP is used as the activating co-factor (Fig. 6c).

## 5 Discussion

Demonstrating links, if any, between chemical exposures and delayed adverse effects such as the development of chronic diseases is a major challenge. This challenge becomes greater when the disease is a rare disease, which is the case with PBC (prevalence of 35/100 000, with an annual incidence of 2–3/100 000 in the North east of England (James et al., 1999; McNally et al., 2014). Although there is a clear genetic pre-disposition to the likelihood of developing PBC, none of the genetic variants predisposing to PBC are in themselves abnormal (Mells et al., 2011; Cordell et al., 2015) and the odds-ratios associated with the most prominent genetic variants (e.g IL12RB2 with an odds ratio 1.52 [95% CI 1.39–1.67] (Mells et al., 2011) are relatively low. This compares to an odds-ratio for the HLA-B*57:01 polymorphism of 36.62 for flucloxacillin liver injury (Nicoletti et al., 2019).

Several studies have identified associations between an environmental factor and PBC incidence (Triger, 1980; Prince et al., 2001; Ala et al., 2006). However, these kind of studies are unlikely to identify individual causative factors (be they chemicals or other factors such as an infection). Effect-directed analysis is a rapidly expanding field of research to identify chemicals in environmental mixtures with toxic effects, using a combination of bioassays and chemical analysis (Brack et al., 2019). The study by Probert et al. (2018) represents an effect-directed analysis with regard to identifying causative agents (or modulating agents) in environmental samples responsible for chronic diseases. Through identifying factors that could be a hazard for triggering a disease (whether they be a realistic risk to the general population or not), we may be able to understand in greater detail the molecular mechanisms of the disease process from the very point of first exposure. With greater understanding of the disease process, and by consideration of related factors (e.g. through read-across), we may be able to predict realistic risks for disease causation. We may also be able to monitor the natural history of the disease process from start to finish in animal models. A major difficulty in studying a disease process such as PBC is that patients are probably identified many years after the trigger process. There may be a significant period where the changes that occur in a PBC patient are uncharacterised. Despite the existence of several spontaneous animal models of PBC-like disease (Katsumi et al., 2015), to date, exposure to a chemical alone has not been demonstrated to trigger a PBC disease-like process.

## 6 Conclusion

Exposure to M8OI (and potentially a range of structurally-related ionic liquids) could result in conversion to a carboxylic acid metabolite in human liver. M8OI may therefore have the potential for replacing lipoic acid in the E2 component of PDC. Oxidative stress associated with the parent M8OI molecule may also contribute to a depletion of lipoic acid and enhance the chances that its carboxylic acid metabolite replace lipoic acid in lipoylated proteins. Such an outcome – in addition to the M8OI parent chemical's ability to induce the apoptosis of liver progenitors and cholangiocytes - could lead to presentation of COOH7IM-modified PDC-E2 to the immune system and loss of tolerance to PDC-E2 and the presence of AMA. However, whether there is any exposure to M8OI (and a range of structurally-related ionic liquids) is not known and presently, it is presumed to be low. Therefore, it remains conjecture that M8OI (and a range of structurally-related ionic liquids) is any risk with respect to triggering PBC.

## Declaration of competing interest

The authors declare that they have no known competing financial interests or personal relationships that could have appeared to influence the work reported in this paper.

## Appendix A Supplementary data

Application Application
